# Gymnasiums for Girls

**Published:** 1889-12

**Authors:** 


### GYMNASIUMS FOR GIRLS.

How much less essential to perfect manhood and womanhood is physical training than education? How much more important is it that scientific rules and principles of health should be observed in the rearing of boys than in the upbringing of girls? These are questions practically intelligent people have come to consider with a seriousness that is making seem foolish many of the theories and superstitions that have hindered the progress of the race and giving the aspect of crimes to certain sins of ommission that stupid custom had sanctioned. There is profound wisdom in the reflections of a discerning writer who says: "As intelligence advances mankind will perceive as a truth of universal application that which is now but a struggling conception on the part of a few who belong to the noble army of fanatics and heretics, viz.: That physical inadequacy is at the root of far more of the vices and sins of to-day than moral delinquency and a systematic intention of doing wrong." The proposition is almost self evident. Physical infirmities and deficiencies resulting from a disregard of the commonest rules of muscular development and bodily perfection must have their reflex in the spiritual and moral character of the victim. Strong, active physical health is rarely associated with moral perversity in the rightly educated man or woman. In a vague way this fact is recognized by parents in its masculine bearing, but an hereditary prejudice against whatever may tend to make girls less delicate, less dainty, than is consistent with the notion of feminine refinement, condemns woman to a position that is in most ways disadvantageous to her highest and best interests.

The terrifying thought that physical training will make hoydens of their ethereal daughters causes most mothers to look frowningly upon the gymnasium. They care to cultivate only those parlor graces that a narrow society has chosen to regard as the evidence of gentility, indifferent that they represent, generally, an inferior state of womanhood. Everything in the discipline of a girl is repressive, bodily and mental, for it is not to be supposed a strong, broad, full mind can find place in a cramped, compressed, stunted physical frame. Free circulation of wholesome blood, the easy, unobstructed play of carefully exercised muscles, are essential to a healthy nervous system and to an active, energetic brain. The young form yet expanding, eager to expand into health and strength, is viciously imprisoned in tight-laced stays, in oppressive clothing, and instead of the vigorous physique shown by the brother, starting with no greater advantages, the girl soon presents a frail, delicate body, many of the functions of which are peremptorily stopped, and struggles into maturity ready to suffer the manifold ills that stubborn prejudice has made her inevitable tormentors. This is because a low idea of what constitutes feminine beauty has made deformity preferable to symmetry, has substituted the taper waist and narrow shoulders and flat chest for the perfect, deep-respiring torse, with its every curve and uniform development. Common sense will rectify in time this egregious blunder, and the classic appreciation of beauty will revive in all its nobility. Just now we are moving slowly and reluctantly in the right direction. It is true the notion of a gymnasium for girls is yet hardly formed into a rational idea, a petty sort of calisthenics that does well for exhibition purposes being its extent. But the proper discipline under competent direction, the limbs unobstructed, and the body allowed free play, is the problem of the future. The dressing is a matter of as great importance as the system of muscular development. Dress reform is possibly keeping pace with the increasing interest in physical culture, and it is gratifying to know there are several schools in this country where girls in sensible costumes are daily exercised in the gymnasium as a part of their general education. These schools are not only successful in themselves, but they have set examples that are being gradually adopted in all institutions for the education of girls and young women. The results are such that it will be astonishing if the ridiculous prejudice against physical training for girls shall not have entirely disappeared within the next twenty years, in this country at least.—*Chicago Inter-Ocean.*

